# Erratum: Skowron Volponi, M. A Vivid Orange New Genus and Species of Braconid-Mimicking Clearwing Moth (Lepidoptera: Sesiidae) Found Puddling on Plecoptera Exuviae. *Insects* 2020, *11*, 425

**DOI:** 10.3390/insects11080518

**Published:** 2020-08-10

**Authors:** Marta Skowron Volponi

**Affiliations:** 1 ClearWing Foundation for Biodiversity, 01-866 Warsaw, Poland; m.skowronvolponi@uwb.edu.pl; 2 Laboratory of Insect Evolutionary Biology and Ecology, Faculty of Biology, University of Bialystok, 15-245 Białystok, Poland

The author wishes to make the following corrections to this paper [1]:

## References

[1] Skowron Volponi M. (2020). A Vivid Orange New Genus and Species of Braconid-Mimicking Clearwing Moth (Lepidoptera: Sesiidae) Found Puddling on Plecoptera Exuviae. Insects.

